# Account of a New Anæsthetic Agent, as a Substitute for Sulphuric Ether in Surgery and Midwifery

**Published:** 1848-02

**Authors:** 


Art. IV.—*Account of a new Anæsthetic Agent, as a Substitute for Sulphuric Ether in Surgery and Midwifery.* By J. Y. Simpson, M.D., F.R.S.E., Prof. Mid. Univ. Edinburgh, &c.

Professor Simpson has introduced to the profession a new agent for inducing insensibility, which bids fair to supersede the ether, and to become a prominent article of the materia medica. Our own experience enables us to speak most favorably of this new anæsthetic agent, and we avail ourselves of all the space at our disposal in this number of our Journal to communicate the results of Professor Simpson's experiments with it. We have inhaled the chloroform ourselves, and administered it to a number of our friends; it has been applied at the Marine Hospital, in a case of delirium tremens, and by Professor Gross in several surgical operations; and uniformly, thus far, its action has been found all that could be desired. We shall have more to say on the subject in our next number, and meantime make room for Prof. S.'s account of this interesting discovery:

"From the time at which I first saw ether inhalation successfully practiced in January last, I have had the conviction impressed upon my mind, that we would ultimately find that other therapeutic agents were capable of being introduced with equal rapidity and success into the system, through the same extensive and powerful channel of pulmonary absorption. In some observations, which I wrote and published in February last, relative to the inhalation of sulphuric ether in midwifery, I stated that, in several obstetric cases, I had used ergot of rye in this way, along with ether.—*See Monthly Journal of Medical Science*, pp. 724 and 795. Case of Successful Inhalation of Opium, to Arrest the Vomiting of Pregnancy.

"With various professional friends, more conversant with chemistry than I am, I have, since that time, taken opportuties of talking over the idea which I entertained of the probable existence or discovery of new therapeutic agents, capable of being introduced into the system by respiration, and the possibility of producing for inhalation vaporizable or volatile preparations of some of our more active and old established medicines: and I have had, during the summer and autumn, ethereal tinctures, etc., of several potent drugs, manufactured for me, for experiment, by Messrs. Duncan, Flockhart, & Co., the excellent chemists and durggists of this city.

"Latterly, in order to avoid, if possible, some of the inconveniences and objections pertaining to sulphuric ether,—(particularly its disagreeable and very persistent smell, its occasional tendency to irritation of the bronchi during its first inspirations, and the large quantity of it occasionally required to be used, more especially in protracted cases of labor,)—I have tried upon myself and others the inhalation of different other volatile fluids, with the hope that some one of them might be found to possess the aduantages of ether, without its disadvantages. For this purpose, I selected for experiment, and have inhaled several chemical liquids of a more fragrant or agreeable odor, such as the chloride of hydrocarbon (or Dutch liquid), acetone, nitrate of oxide of ethyle (nitric ether), benzoin, the vapor of iodoform, etc. I have found, however, one infinitely more efficacious than any of the others, viz: chloroform, or the perchloride of formyl, and I am enabled to speak most confidently of its superior anæsthetic properties, having now tried it upon upwards of thirty individuals. The liquid I have used has been manufactured for me by Mr. Hunter, in the laboratory of Messrs. Duncan, Flockhart & Co.

Chloroform was first discovered and described at nearly the same time by Soubeiran (1831), and Liebig (1832); its composition was first accurately ascertained by the distinguished French chemist Dumas, in 1835.—See the *Annales de Chimie et de Physique*, vols. xlviii., xlix., and lviii. It has been used by some practitioners internally; Guillot prescribed it as an anti-spasmodic in asthma, exhibiting it in small doses, and diluted 100 times. (See Bouchardat's *Annuaire de Therapeutique*, for 1844, p. 35.) But no person, so far as I am aware, has used it by inhalation, or discovered its remarkable anæsthetic properties till the date of my own experiments.

"It is a dense, limpid, colorless liquid, readily evaporating, and possessing an agreeable, fragrant, fruit-like odor, and a saccharine pleasant taste.

"As an inhaled anæsthetic agent, it possesses over suphuric ether the following advantages:

"1. A greatly less quantity of chloroform than of ether is requisite to produce the anæsthetic effect; usually from a hundred to a hundred and twenty drops of chloroform only being sufficient; and with some patients much less. I have seen a strong person rendered completely insensible by six or seven inspirations of thirty drops of the liquid.

"2. Its action is much more rapid and complete, and generally more persistent. I have almost always seen from ten to twenty full inspirations suffice. Hence the time of the surgeon is saved; and that preliminary stage of excitement, which pertains to all narcotizing agents, being curtailed, or indeed practically abolished, the patient has not the same degree of tendency to exhilaration and talking.

"3. Most of those who know from previous experience the sensations produced by ether inhalation, and who have subsequently breathed the chloroform, have strongly declared the inhalation and influence of chloroform to be far more agreeable and pleasant than those of ether.

"4. I believe, that considering the small quantity requisite, as compared with ether, the use of chloroform will be less expensive than that of ether; more especially, as there is every prospect that the means of forming it may be simplified and cheapened.

"5. Its perfume is not unpleasant, but the reverse; and the odor of it does not remain, for any length of time, obstinately attached to the clothes of the attendant,—or exhaling in a disagreeable form from the lungs of the patient, as so generally happens with sulphuric ether.

"6. Being required in much less quantity, it is much more portable and transmissible than sulphuric ether.

"7. No special kind of inhaler or instrument is necessary for its exhibition. A little of the liquid diffused upon the interior of a hollow-shaped sponge, or a pocket-handkerchief, or a piece of linen or paper, and held over the mouth and nostrils, so as to be fully inhaled, generally suffices in about a minute or two to produce the desired effect.

"I have not had an opportunity of using chloroform in any capital surgical operation, but have exhibited it, with perfect success, in tooth-drawing, opening abscesses, for annulling the pain of dysmenorrhea and of neuralgia, and in two or three cases where I was using deep and otherwise very painful galvano-puncture for the treatment of ovarian dropsy, etc. I have employed it also in obstetric practice with entire success.

"The lady to whom it was first exhibited during parturition, had been previously delivered in the country by perforation of the head of the infant, after a labor of three days' duration. In this, her second confinement, pains supervened a fortnight before the full time. Three hours and a half after they commenced, and, ere the first stage of the labor was completed, I placed her under the influence of the chloroform, by moistening, with half a teaspoonful of the liquid, a pocket-handkerchief, rolled up into a funnel shape, and with the broad or open end of the funnel placed over her mouth and nostrils. In consequence of the evaporation of the fluid, it was once more renewed in about ten or twelve minutes. The child was expelled in about twenty-five minutes after the inhalation was begun. The mother subsequently remained longer soporose than commonly happens after ether. The squalling of the child did not, as usual, rouse her; and some minutes elapsed after the placenta was expelled, and after the child was removed by the nurse into another room, before the patient awoke. She then turned round and observed to me that she had "enjoyed a very comfortable sleep, and indeed required it, as she was so tired, but would now be more able for the work before her." I evaded entering into conversation with her, believing, as I have already stated, that the most complete possible quietude forms one of the principal secrets for the successful employment of either ether or chloroform. In a little time she again remarked that she was afraid her "sleep had stopped the pains." Shortly afterwards, her infant was brought in by the nurse from the adjoining room, and it was a matter of no small difficulty to convince the astonished mother that the labor was entirely over, and that the child presented to her was really her "own living baby."

"Perhaps I may be excused from adding, that since publishing on the subject of ether inhalation in midwifery, seven or eight months ago, and then for the first time directing the attention of the medical profession to its great use and importance in natural and morbid parturition, I have employed it, with few and rare exceptions, in every case of labor that I have attended; and with the most delightful results. And I have no doubt whatever, that some years hence the practice will be general. Obstetricians may oppose it, but I believe our patients themselves will force the use of it upon the profession. I have never had the pleasure of watching over a series of better and more rapid recoveries; nor once witnessed any disagreeable result follow to either mother or child; whilst I have now seen an immense amount of maternal pain and agony saved by its employment. And I most conscientiously believe that the proud mission of the physician is distinctly twofold—namely, to alleviate human suffering, as well as to preserve human life."

The following is an eligible formula for preparing the chloroform:

| | | |
|---|---|---|
| "℞ | Chloride of lime in powder, | ℔iv. |
| | Water, - - - - | ℔xii. |
| | Rectified Spirit, - - - | f ℥xii. |

Mix in a capacious retort or still, and distill as long as a dense liquid, which sinks in the water with which it comes over, is produced."

